# The EAR Motif Controls the Early Flowering and Senescence Phenotype Mediated by Over-Expression of SlERF36 and Is Partly Responsible for Changes in Stomatal Density and Photosynthesis

**DOI:** 10.1371/journal.pone.0101995

**Published:** 2014-07-18

**Authors:** Rakesh Kumar Upadhyay, Asmita Gupta, Sanjay Ranjan, Ruchi Singh, Uday V. Pathre, Pravendra Nath, Aniruddha P. Sane

**Affiliations:** 1 Plant Gene Expression Lab, CSIR-National Botanical Research Institute (Council of Scientific and Industrial Research), Lucknow, India; 2 Department of Plant Physiology, CSIR-National Botanical Research Institute (Council of Scientific and Industrial Research), Lucknow, India; 3 Sustainable Agricultural Systems Laboratory, USDA-ARS, Beltsville Agricultural Research Center, Beltsville, Maryland, United States of America; 4 Department of Biology, Pennsylvania State University, Harrisburg, Pennsylvania, United States of America; University of Leeds, United Kingdom

## Abstract

The EAR motif is a small seven amino acid motif associated with active repression of several target genes. We had previously identified *SlERF36* as an EAR motif containing gene from tomato and shown that its over-expression results in early flowering and senescence and a 25–35% reduction of stomatal density, photosynthesis and stomatal conductance in transgenic tobacco. In order to understand the role of the EAR motif in governing the phenotypes, we have expressed the full-length *SlERF36* and a truncated form, lacking the EAR motif under the CaMV35S promoter, in transgenic Arabidopsis. Plants over-expressing the full-length *SlERF36* show prominent early flowering under long day as well as short day conditions. The early flowering leads to an earlier onset of senescence in these transgenic plants which in turn reduces vegetative growth, affecting rosette, flower and silique sizes. Stomatal number is reduced by 38–39% while photosynthesis and stomatal conductance decrease by about 30–40%. Transgenic plants over-expressing the truncated version of SlERF36 (lacking the C-terminal EAR motif), show phenotypes largely matching the control with normal flowering and senescence indicating that the early flowering and senescence is governed by the EAR motif. On the other hand, photosynthetic rates and stomatal number were also reduced in plants expressing SlERF36ΔEAR although to a lesser degree compared to the full- length version indicating that these are partly controlled by the EAR motif. These studies show that the major phenotypic changes in plant growth caused by over-expression of SlERF36 are actually mediated by the EAR motif.

## Introduction

The AP2-ERF (APETALA2-ETHYLENE RESPONSE FACTOR) domain family of transcription factors is one of the largest families of transcription factors comprising about 140–280 members in various plants [1]. The family governs plant responses to various biotic and abiotic stresses largely by controlling responses to different plant hormones such as ABA, ethylene and jasmonic acid (JA). A small sub set of this family is characterized by the presence of a seven amino acid repression motif designated as the ERF-associated amphiphilic repression (EAR) motif that is present at the C-terminal end of the protein. The EAR motif with an L/FDLNL/FxP sequence functions in concert with the AP2 domain but is not restricted to the ERF family. It has been found to be associated with about 21 different types of transcriptional regulator families that also include the AUX/IAA family (with the similar LxLxL motif), C2H2 Zn finger family and the JAZ family [2], [3]. The domain can confer the ability to repress transcription even when it is hooked on to proteins that otherwise function as transcriptional activators [4], [5]. The EAR motif and the related LxLxL motif actively repress target genes by recruitment of co-repressors such as AtSAP18/SIN3 [6], [7] or those of the TOPLESS family such as TPL/TPR [8]. These co-repressors in turn interact with histone deacetylases and remodel chromatin to repress transcription [9], [3]. The presence of the EAR motif is essential for the repression activity with mutations in the D and L residues of the motif affecting the repressor function [4], [10], [11].

At least eight EAR motif containing ERF genes are known in Arabidopsis and rice [1] and at least seven are present in tomato [12], [13]. They show diverse roles such as in herbivory and wounding [14], [15], cold and drought stresses [16], salt stress signalling [17], ABA responses [6] ethylene response [13], [18], [19], JA responses [20] and senescence [21].While the EAR motif has been shown to function as a repressor motif *in vitro*, its role and the extent of its contribution in governing the processes mediated by its expression are still not clear. We had previously identified SlERF36/SlERF.F.1 (Accession No. SGN-U564952) as an EAR motif containing AP2 domain gene from tomato, the expression of which accelerated flowering and senescence, and reduced stomatal density by 25–35% in transgenic tobacco plants with direct or indirect effects on photosynthesis, stomatal conductance and transpiration [22]. In this paper, we show that the phenotypic effects of early flowering and senescence imparted by *SlERF36* over-expression are largely governed by the EAR motif while the reduction in stomatal density appears to be partly dependent on the EAR motif.

## Materials and Methods

### Development of constructs and transgenic *Arabidopsis* plants

The SlERF36 gene was identified in a previous study as an EAR motif containing AP2 domain gene encoding a protein of 221 amino acids [22]. Constructs containing the SlERF36 gene under the CaMV35S promoter in a pBI121 background (used for transgenic tobacco plants [22]) were also used to transform the Col-0 ecotype of *Arabidopsis*. In order to study the effects of lack of EAR repressor motif, a reverse primer SlERF36ΔEAR-R GTCAAGGCAGTGGATTTCTGAGAGATGA was designed just upstream of the EAR motif that incorporated a termination codon. This primer was used in combination with the forward primer ATGGATCCTATGAGAAGAGGCAGAGC (containing the initiation codon) to amplify a fragment of 598 nucleotides that contained an ORF of 585 nt. This ORF encoded a protein of 195 amino acids and lacked the last 26 amino acids that also included the EAR motif. The fragment was cloned in pBI121 under the CaMV35S promoter. *Arabidopsis* plants were transformed with these constructs using the floral dip method [23].

Of the various transformants generated, two each (lines 1-8 and 2-1 for SlERF36 and lines 6-1 and 9-1 for SlERF36ΔEAR, lacking the EAR motif) were taken to the third generation to obtain homozygous lines. Progeny of these homozygous lines was used for all phenotypic and physiological studies as well as for gene expression. The plants were grown in culture racks in cups in soilrite mix at a light intensity of 150 µmoles and a photoperiod of 16 h light/8 h dark at 22°C unless otherwise mentioned. For short day treatments plants were grown in a 10 h light/14 h dark cycle. The plants were monitored and compared for various growth parameters that included leaf number, rosette diameter, time of flowering, time of senescence, chlorophyll content, plant height, flower and silique sizes as well as photosynthetic parameters and stomatal density.

### Gene expression

Gene expression studies were carried out using RNA isolated from seedlings of control (Col-0) and transgenic plants (with and without the EAR motif). RNA was isolated using the Spectrum Plant total RNA isolation kit (Sigma) and cDNA prepared using the REVERTAID MMLV kit (Fermentas). Expression of *SlERF36* in the transgenic lines was carried out using the primers SlERF36-FRT: TTCCGTCGTTGACCACGGCG and SlERF36-RRT: TGCAGTGAAGA TCGTCGGCGC and normalized against actin amplified using the primers Act-F: ATGACATGGAGAAGATCTGGCATCA and Act-R: AGCCTGGATGGCAACATACAT AGC. Analysis of FT transcript levels was carried out using the primers FT-F: TGTTGGAGACGTTCTTGATCC and FT-R: AGCCACTCTCCCTCTGACAA [24] while analysis of SEN4 was carried out using the primers SEN4-F: TCTTCTTCACGACTCTTCTC and SEN4-R: TTGCCCAATCGTCTGCGTTC [25].

### Physiological studies

Homozygous *Arabidopsis* plants expressing *SlERF36* and *SlERF36 ΔEAR* under the CaMV35S promoter were grown in pots in the culture room under white light. Progeny of homozygous lines was used for each experiment with 5–6 plants per line. Gas exchange parameters were determined on plants enclosed in an *Arabidopsis* chamber (3010-A) under controlled conditions using the GFS-3000 (Heinz Walz Gmbh, Effeltrich, Germany) instrument attached with a fluorescence module (LED-array/PAM fluorometer 3055-FL, Walz, Germany). Measurements were made on 20-day-old plants. Measurements of steady-state photosynthesis rate (A), stomatal conductance (g_s_) and transpiration rate (E) were carried out at 70% humidity, 25°C leaf temperature, CO_2_ concentration of 400 µmol mol^−1^and PPFD adjusted to 400 µmol photons m^−2^s^−1^ as described before [22].

Chlorophyll content was measured by isolating chlorophyll from leaves of 12-day-old plants (4^th^ leaf from bottom) and calculated according to Arnon [26].

### Estimation of stomatal density

Leaf epidermis (about 1 cm^2^) from the abaxial surface of fully expanded leaves (7^th^ leaf from bottom of 30-day-old plants) was peeled off with a pair of forceps and placed immediately in water and later mounted in 10% glycerol and observed under a light microscope (Nikon Eclipse TE300 Inverted microscope). Stomata were counted in an area of 0.24 µm^2^ in three different regions from three independent leaves of three plants from the same position from two independent lines (1-8 and 2-1 expressing *SlERF36* and 6-1 and 9-1 expressing *SlERF36ΔEAR*).

### Statistical Analysis

Stastitical analysis for all growth parameters and stomatal numbers was carried out using Student's t-test with *P* values of <0.05 considered statistically significant. For physiological parameters, the significance of correlations was tested by using linear regression, with *P* values of <0.05 considered statistically significant. Means were compared by using one-way analysis of variance and post hoc means comparison (Scheffé Test). Data analysis and plotting were performed with SigmaPlot version 8 0.

## Results

**Figure 1:**
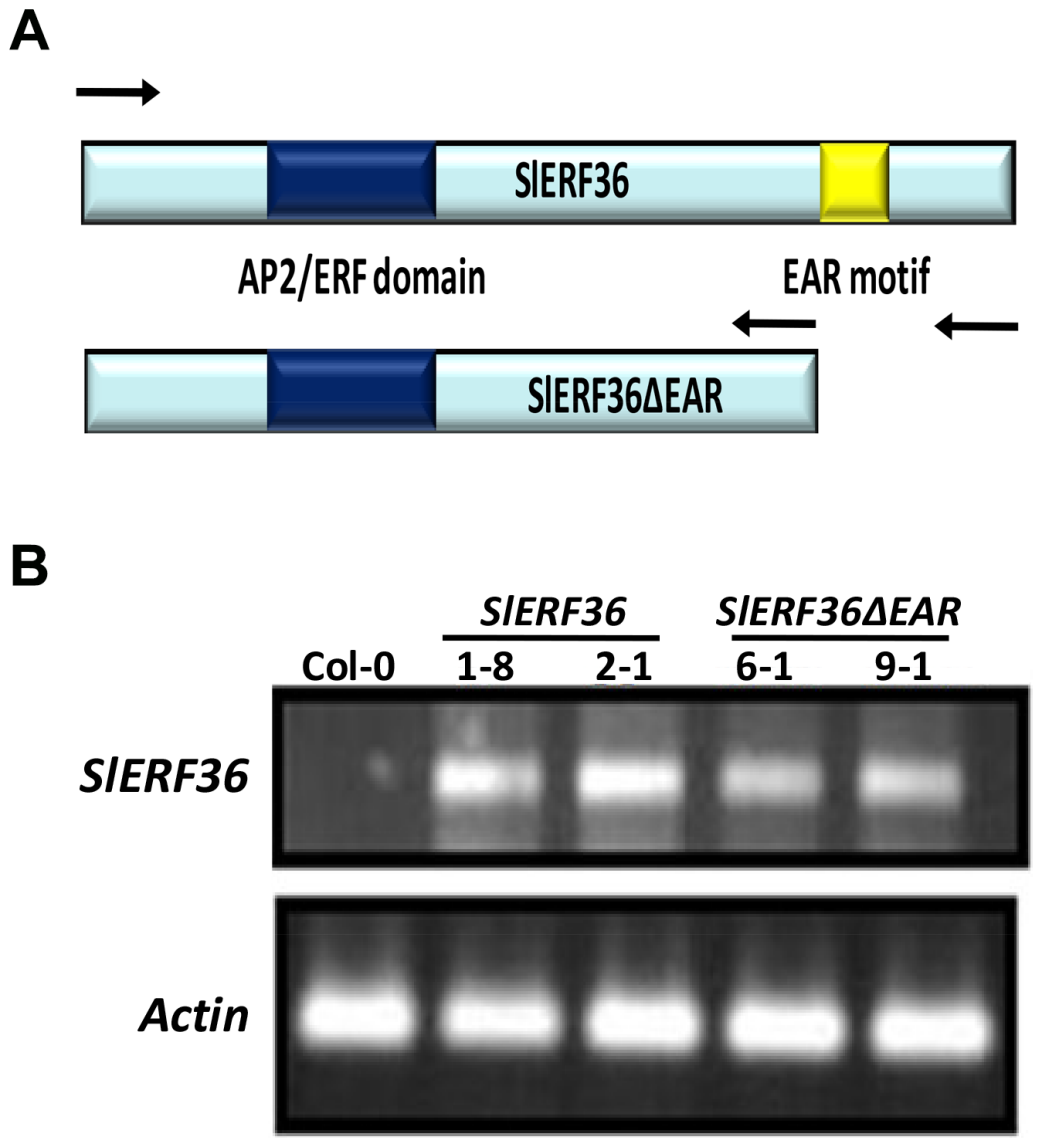
The full length and truncated versions of SlERF36 used for study. **A.** Schematic representation of SlERF36 and SlERF36ΔEAR that lacks the C-terminal region. The EAR motif is shown in yellow while the AP2 domain has been shown in dark blue. The arrows represent the primers used to amplify the two genes. **B.** Expression of the transgenes *SlERF36* and *SlERF36ΔEAR* in the transgenic lines 1-8 and 2-1 (*SlERF36*) and the lines 6-1 and 9-1 (*SlERF36ΔEAR*). Actin was used as a control.

Over-expression of SlERF36/SlERF.F.1 was previously shown to affect flowering time and senescence and was responsible for a 25–35% reduction in stomatal density that affected several photosynthetic parameters in tobacco [22]. In order to perform a more detailed study regarding the role of the EAR motif in these effects, constructs containing the full length *SlERF36* and one lacking the EAR motif (*SlERF36ΔEAR*) were generated for expression under the CaMV35S promoter (Fig. 1A). Both constructs were used to transform *Arabidopsis*. Of the various independent lines generated, two designated as SlERF36-1-8 and SlERF36-2-1 (over-expressing the full length *SlERF36*) and SlERF36ΔEAR-6-1 and SlERF36ΔEAR-9-1 (over-expressing *SlERF36ΔEAR*) were selected for detailed analysis in the third generation. Progenies of homozygous lines were first checked by semi-quantitative RT-PCR (using actin as internal control) with primers specific to a region common to *SlERF36* and *SlERF36ΔEAR* to confirm that the genes were expressed in the respective transgenic lines (Fig. 1B). The plants were then monitored for various visible growth parameters such as time of flowering, leaf shape and size, senescence, height, and physiological characters.

### *SlERF36* over-expressing plants show early photoperiod independent flowering

**Figure 2:**
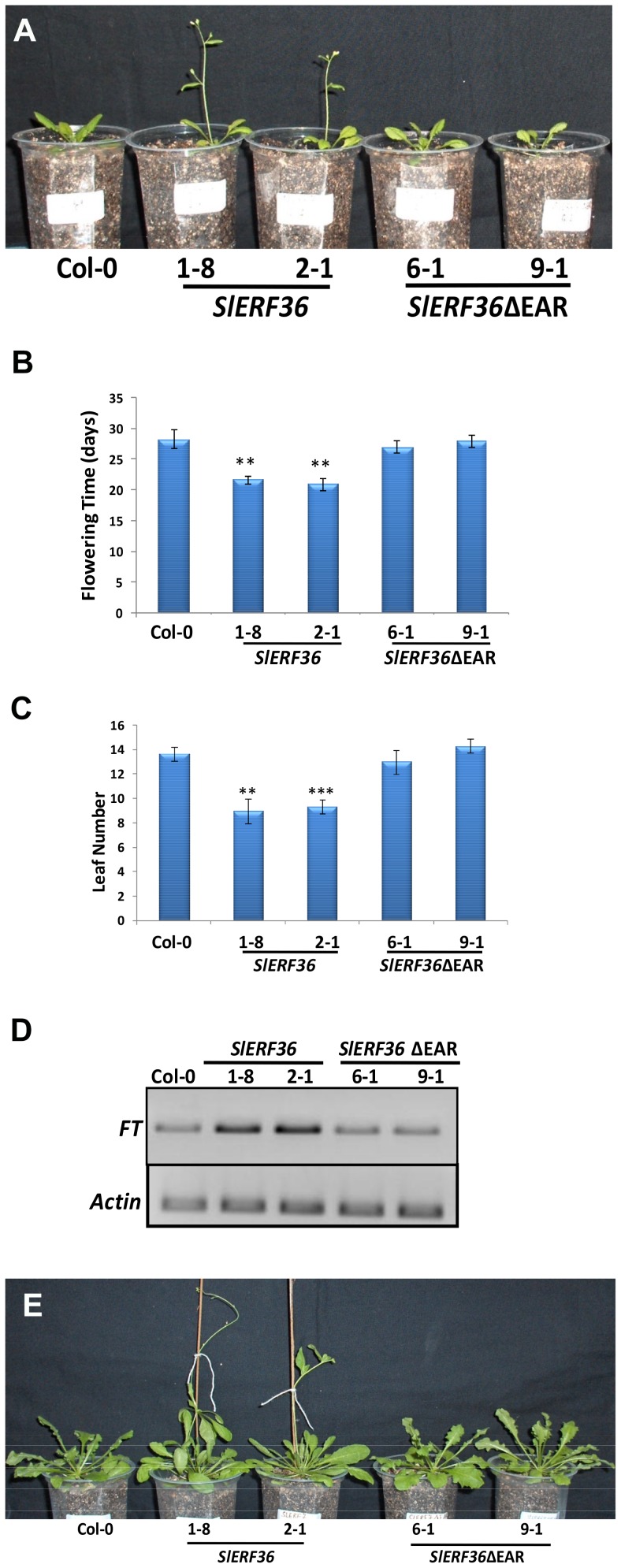
Early flowering in transgenic *SlERF36* over-expressing plants. **A.** Early flowering in transgenic *SlERF36* over-expressing plants grown under long day (16 h light/8 h dark) conditions. Col-0 wild type; Lines 1-8 and 2-1 – *SlERF36* over-expressing lines; Lines 6-1 and 9-1 – *SlERF36ΔEAR* over-expressing lines. **B.** Graphical representation of days to flowering in transgenic *SlERF36* and *SlERF36ΔEAR* over-expressing lines. Values represent the average ± SD of 3–5 homozygous plants of each independent transformant.** P<0.01; ***P<0.001. **C.** Graphical representation of number of leaves at the time of flowering in transgenic *SlERF36* and *SlERF36ΔEAR* over-expressing lines. Values represent the average ± SD of 3–5 homozygous plants of each independent transformant.** P<0.01; ***P<0.001. **D.** Transcript accumulation of the *FT* gene in control and transgenic lines in 12-day-old plants by semi-quantitative RT-PCR. Actin was used as internal control. **E.** Early flowering in transgenic *SlERF36* over-expressing plants grown under short day (10 h light/14 h dark) conditions. Note the delay in flowering and the larger rosettes compared to plants grown in long days in Fig. 3A.

One of the most prominent features of transgenic *SlERF36* over-expressing plants was the early flowering phenotype (Fig. 2A). In comparison to control plants which produced inflorescence bolts at 28.33±1.52 days, transgenic *SlERF36* over-expressing plants produced inflorescence bolts at about 21.66±0.57 days (*SlERF36-*1-8) and 21.0±1.0 days (*SlERF36-*2-1) indicating a decrease in flowering time by about 7 days compared to the control (Fig. 2B). In contrast, transgenic *SlERF36ΔEAR* over-expressing plants lacking the EAR motif, produced inflorescence bolts at 27.0±1.0 days (SlERF36ΔEAR-6-1) and 28.0±1.0 days (SlERF36ΔEAR-9-1).

Flowering in transgenic *SlERF36* plants was initiated with fewer leaves as compared to control and *SlERF36ΔEAR* (Fig. 2C). Control plants had an average number of 13.66±0.57 leaves at the time of flowering while transgenic *SlERF36* over-expressing plants flowered at 9.0±1.00 leaves/plant (SlERF36-1-8) to 9.33±0.57 leaves/plant (SlERF36*-*2-1). This early flowering phenotype of *SlERF36* over-expressing plants was no longer observed after deletion of the C-terminal region containing the repressor domain. Transgenic *SlERF36ΔEAR* over-expressing plants flowered at 13.0±1.00 leaves/plant (*SlERF36ΔEAR*-6-1) to 14.33±0.57 leaves/plant (*SlERF36ΔEAR-*9-1).

We next checked for expression levels of *FT* (*FLOWERING LOCUS T*) transcript in 20-day-old plants. As shown in Fig. 2D, a higher transcript level of *FT* was observed in *SlERF36* plants compared to Col-0 and *SlERF36ΔEAR* plants indicating that the presence of the C-terminal region containing the EAR motif was essential for the increase in *FT* levels.

We then tested whether the early flowering phenotype was dependent on photoperiod. For this, plants were grown under 24 h light as well as under short day conditions. Under all conditions, flowering was earlier in *SlERF36* over-expressing plants as compared to control and *SlERF36ΔEAR* over-expressing plants. Under a 24 h light period, flowering was advanced by about 10 days in transgenic *SlERF36* plants while under short day conditions it was advanced by one and half months compared to control and *SlERF36ΔEAR* plants (Fig. 2E) indicating that the early flowering conferred by SlERF36 was independent of photoperiod.

### *SlERF36* over-expressing plants show early senescence

**Figure 3:**
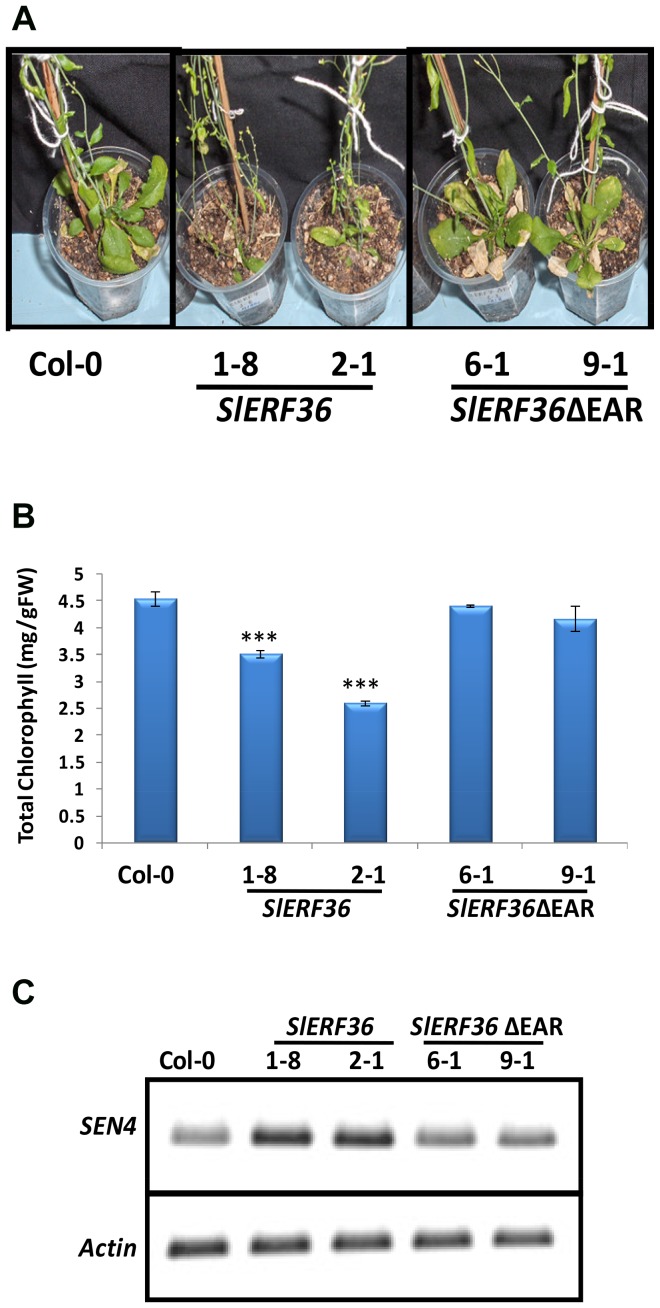
Early senescence in transgenic *SlERF36* over-expressing plants. One and half month old plants (grown under long day conditions from Fig. 3A) showing early leaf senescence and death. Graphical representation of differences in chlorophyll content between control, *SlERF36* over-expressing (1-8 and 2-1) and *SlERF36ΔEAR* over-expressing (6-1 and 9-1) plants. Values represent the average ± SD of 5 leaves of each independent transformant (4^th^ leaf from bottom from 12-day-old plants). Transcript accumulation of the senescence associated *SEN4* gene in control and transgenic lines in 12-day-old plants. Actin was used for normalization.

The initiation of flowering was followed by rapid senescence and leaf death in transgenic *SlERF36* over-expressing lines as shown in Fig. 3A. The early onset of senescence manifested itself in the form of reduced chlorophyll content, early yellowing and early death in lower leaves of transgenic *SlERF36* over-expressing lines as compared to the controls (Fig. 3A and B). At the 12-day-stage, control plants (fourth leaf from bottom) had a chlorophyll content of 4.55±0.13 mg/g FW. In contrast, the leaves of transgenic plants of *SlERF36* over-expressing lines had a chlorophyll content of 3.51±0.07 mg/g FW (*SlERF36-*1-8) and 2.60±0.05 mg/g FW (*SlERF36-*2-1) indicating a decrease in chlorophyll content by 25–50% of the control (Fig. 3B). In contrast, transgenic *SlERF36*ΔEAR over-expressing plants showed normal wild type senescence with chlorophyll content ranging from a minimum of 4.17±0.23 mg/g FW (*SlERF36ΔEAR-*9-1) to 4.42±0.02 mg/g FW (*SlERF36ΔEAR-*6-1). The early senescence in *SlERF36* expressing plants was also evident at the molecular level by higher expression of the *SEN4* gene (a marker gene for senescence)[25] (Fig. 3C).

**Figure 4:**
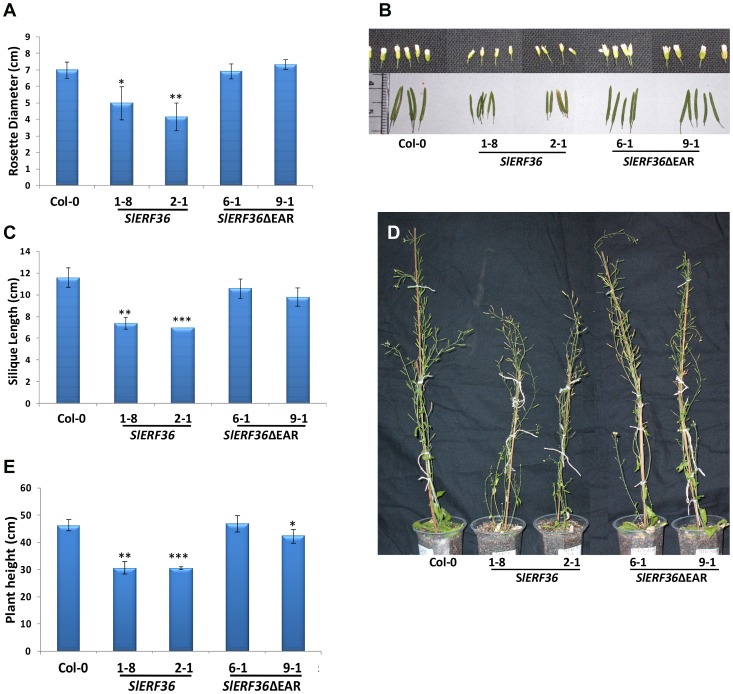
Reduction in organ size and plant height of transgenic *SlERF36* over-expressing plants grown under long day conditions. A. Graphical representation of rosette diameter of 28-day-old transgenic plants over-expressing *SlERF36* and *SlERF36ΔEAR*. Values represent the average ± SD of 3–5 homozygous plants of each independent transformant.** P<0.01; ***P<0.001. B. Flower (top) and silique (bottom) size variation in transgenic *SlERF36* and *SlERF36*ΔEAR *Arabidopsis* plants. C. Graphical representation of the variation in silique sizes in transgenic *SlERF36* and *SlERF36*ΔEAR *Arabidopsis* plants. Values represent the average ± SD of 10 siliques each from 3–5 homozygous plants of each independent transformant.** P<0.01; ***P<0.001. D. Comparison of transgenic *SlERF36* and *SlERF36*ΔEAR over-expressing plants showing differences in height. E. Graphical representation of plant height of transgenic *SlERF36* and *SlERF36*ΔEAR plants. Values represent the average ± SD of 3–5 homozygous plants of each independent transformant. ** P<0.01; ***P<0.001.

The early flowering phenotype markedly affected plant growth, height and flower and silique length. In general, transgenic *SlERF36* over-expressing plants had a smaller rosette diameter as compared to control Col-0 and *SlERF36*ΔEAR over-expressing plants (Fig. 4A). Rosettes measured in 28-day-old plants had a diameter of 7.0±0.5 cm for control plants. In contrast the rosette diameter of transgenic plants of *SlERF36* over-expressing lines ranged from a minimum of 4.1±0.85 cm (*SlERF36 -*2-1) to 5.0±1.0 cm (*SlERF36 -*1-8) indicating a decrease by 2–3 cm in diameter compared to the control. Deletion of the repressor domain led to formation of normal rosettes with size ranges from 6.9±0.45 cm (*SlERF36*ΔEAR*-*6-1) to 7.33±0.28 cm (*SlERF36*ΔEAR*-*9-1).

Transgenic *SlERF36* over-expressing plants also had smaller flowers and siliques as compared to control Col-0 and *SlERF36ΔEAR* over-expressing plants (Fig. 4B). As compared to a silique length of 11.6±0.89 cm in controls, transgenic *SlERF36* over-expressing plants showed siliques of sizes 7.4±0.54 cm (*SlERF36-*1-8) and 7.0±0.0 cm (*SlERF36-*2-1). In contrast, transgenic *SlERF36ΔEAR* over-expressing plants had siliques ranging in length from 9.8±0.83 cm (*SlERF36ΔEAR-*9-1) to 10.6±0.89 cm (*SlERF36ΔEAR-*6-1) (Fig. 4C). Full grown plants over-expressing *SlERF36* were shorter as compared to control Col-0 and *SlERF36ΔEAR* over-expressing plants (Fig. 4D) with a final plant height of 30.66±2.30 cm (*SlERF36-*1-8) and 30.70±0.60 cm (*SlERF36*-2-1) as compared to about 46.33±2.08 cm in controls and 47.00±3.0 cm (*SlERF36ΔEAR*-6-1) and 44.83±5.29 cm (*SlERF36ΔEAR*-9-1). This indicated a decrease in height by 35–40% for 35S:SlERF36 plants compared to that of the control and transgenic 35S:*SlERF36*ΔEAR plants.

### Transgenic 35S:SlERF36 *Arabidopsis* plants show reduced photosynthesis and stomatal conductance

**Figure 5:**
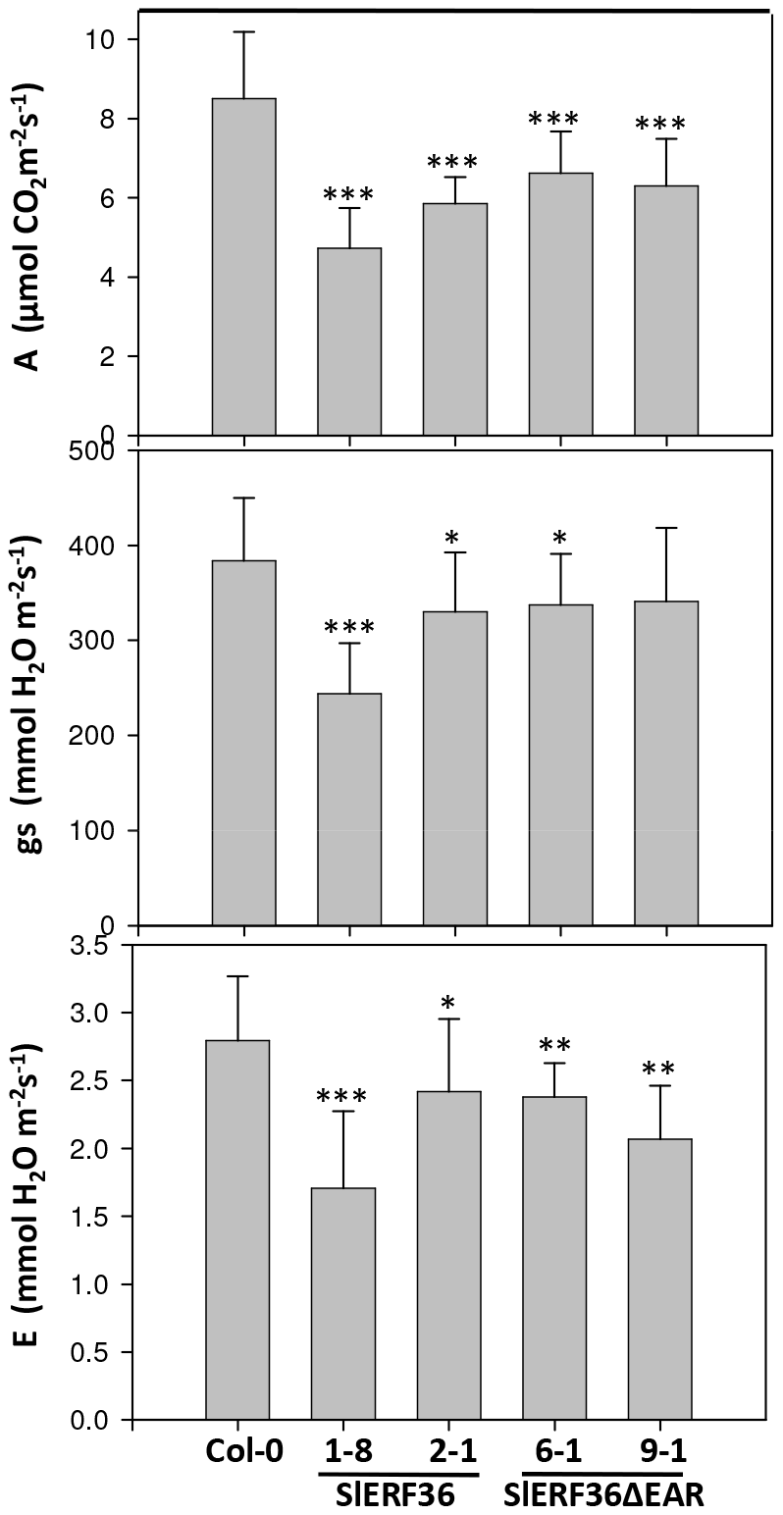
Net photosynthesis (A), stomatal conductance (gs) and transpiration (E) rates of transgenic *Arabidopsis* plants over-expressing *SlERF36* and *SlERF36ΔEAR*. Five plants of homozygous progeny of two independent lines for each gene (lines 1-8 and 2-1 over-expressing *SlERF36* and lines 6-1 and 9-1 over-expressing *SlERF36ΔEAR*) were grown as described in the methods section. Measurements were carried out using a GFS-3000 system under a light intensity of 400 µmole photons m^−2^s^−1^ and a CO_2_ concentration of 400 µmol mol^−1^. Values are average ± SEs of five replicates. *P<0.05, ** P<0.01; ***P<0.001, ****P<0.0001.

To study if reduced growth and early senescence was due to an effect on photosynthesis we carried out a detailed analysis of the various photosynthetic parameters of the transgenic lines using a GFS-3000 system. As shown in Fig. 5, photosynthetic rates showed a marked reduction in transgenic lines. Compared to control plants that showed photosynthetic rates of 8.75±1.24 µmol CO_2_ fixed m^−2^s^−1^ plants over-expressing *SlERF36* showed reduced photosynthetic rates ranging from 4.8±1.1 (*SlERF36*-1-8) to 5.9±0.54 µmol CO_2_ fixed m^−2^s^−1^ (*SlERF36*-2-1). This indicated a reduction of 33–45% compared to the control. Interestingly, the reduction in photosynthetic rates brought about by over-expression of *SlERF36* was partially affected by the removal of the EAR motif with transgenic *SlERF36ΔEAR* over-expressing lines showing rates of 6.4±1.26 (*SlERF36ΔEAR*-6-1) to 6.1±1.24 µmol CO_2_ fixed m^−2^s^−1^ (*SlERF36ΔEAR*-9-1) indicating a reduction of 27–30% compared to control. The reduction in photosynthetic rates was associated with a decrease in stomatal conductance and transpiration in transgenic lines. Stomatal conductance reduced from 385±62 mmol H_2_O m^−2^s^−1^ in control plants to 240±52 (*SlERF36*-1-8) and 323±64 mmol H_2_O m^−2^s^−1^ (*SlERF36*-2-1) in the transgenic lines. A decrease in stomatal conductance was also seen in transgenic *SlERF36ΔEAR* over-expressing lines with values of 332±52 and 340±81 mmol H_2_O m^−2^s^−1^ in lines *SlERF36ΔEAR*-6-1 and *SlERF36ΔEAR*-9-1 respectively. Transpiration also showed a decrease of 14–40% in transgenic *SlERF36* over-expressing lines and a decrease of 14–27% in transgenic *SlERF36ΔEAR* over-expressing lines. No changes were observed in internal Ci concentration, ETR rates, yield and Fv/Fm values (data not shown).

### Transgenic *SlERF36* over-expressing lines show reduced stomatal density

**Figure 6:**
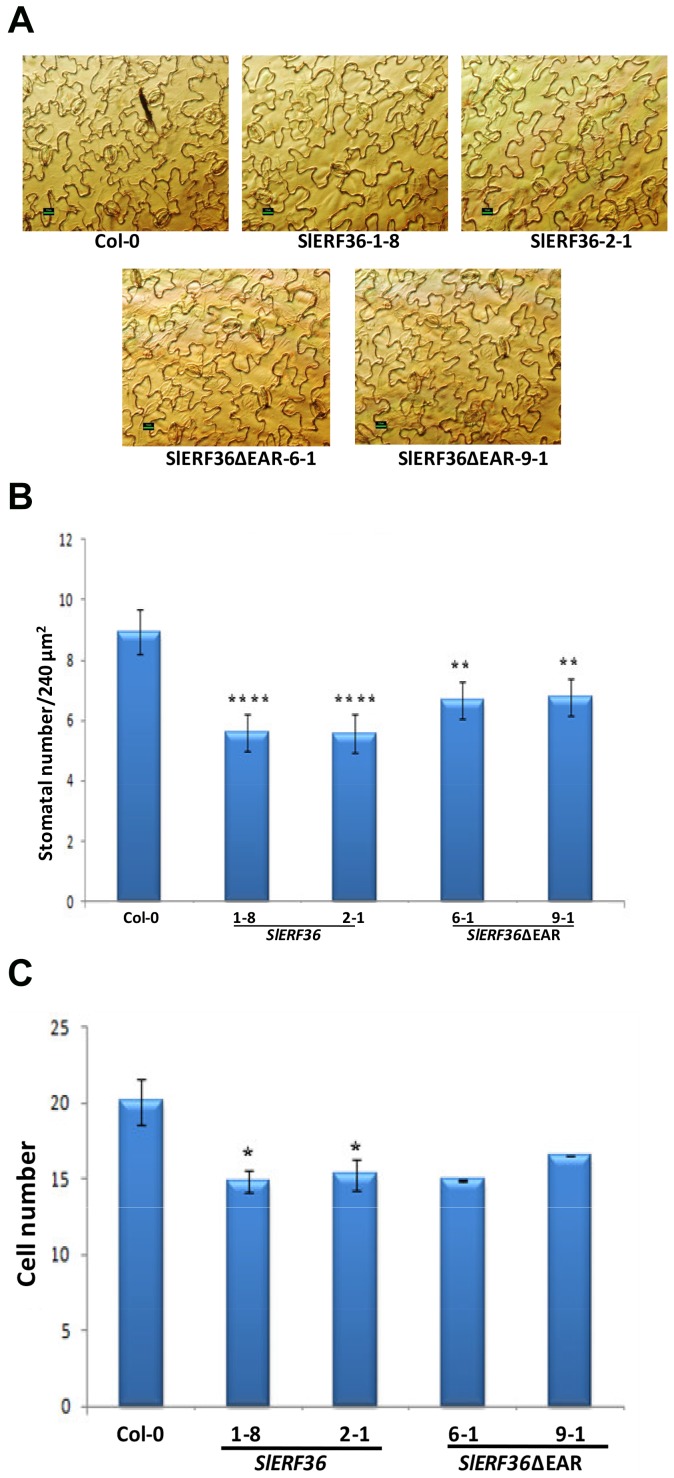
Reduction in stomatal density in transgenic *SlERF36* and *SlERF36ΔEAR* plants. A. Stomatal density on the leaf abaxial surface in control (C) and transgenic *Arabidopsis* plants from two independent lines (lines 1-8 and 2-1 over-expressing *SlERF36* and lines 6-1 and 9-1 over-expressing *SlERF36ΔEAR*). Stomatal density from leaf epidermal peels was estimated in the leaf sections in three different regions of three different leaves (7^th^ leaf from bottom from 30-day-old plants) under a light microscope (Nikon Eclipse TE300 Inverted microscope). The small black bar at the base of each picture on the left hand side represents a length of 10 µm. **B.** Graphical estimation of the stomatal density of the lower leaf epidermis of control (Col-0) and transgenic *SlERF36* and *SlERF36ΔEAR* over-expressing lines from Fig. 6A. Values represent the average stomatal density ± SD in an area of 240 µm^2^ of three independent leaves (from the same position). ** P<0.01; ***P<0.001, ****P<0.0001. Figure 6C. Graphical estimation of the non-stomatal cell number of the lower leaf epidermis of control (Col-0) and transgenic *SlERF36* and *SlERF36ΔEAR* over-expressing lines from Fig. 6A. Values represent the average cell number± SD in an area of 240 µm^2^ of three independent leaves (from the same position). * P<0.05.

Our previous results had shown that over-expression of *SlERF36* affects stomatal density and that many of the defects in photosynthetic parameters were most likely associated with adaptive responses due to reduced stomatal number [22]. To test whether the reduction in photosynthetic parameters was associated with stomatal number, we measured the stomatal density on the abaxial leaf surface (7^th^ leaf from bottom of 30-day-old plants) of control and transgenic lines. As shown in Fig. 6 (A and B) control *Arabidopsis* plants showed a stomatal number of about 8.94±0.76/240 µm^2^. In contrast transgenic *SlERF36* over-expressing lines showed a stomatal number of 5.55±0.61/240 µm^2^ (*SlERF36*-1-8) and 5.61±0.61/240 µm^2^ (*SlERF36*-2-1) in the same field. This indicated a decrease in stomatal number to just 61–62% of the controls in transgenic *SlERF36* over-expressing lines. The removal of the EAR motif in transgenic *SlERF36ΔEAR* over-expressing lines led to a comparative increase in stomatal numbers with values of 6.66±0.6/240 µm^2^ and 6.77±0.64/240 µm^2^ in the lines *SlERF36ΔEAR*-6-1 and *SlERF36ΔEAR*-9-1 respectively. Thus over-expression of the truncated gene lacking the EAR motif affected the stomatal number to a lesser degree with a decrease of 25–26% compared to the decrease of 38–39% in lines expressing the full length *SlERF36* gene. The difference between the stomatal numbers of the *SlERF36* and *SlERF36ΔEAR* over-expressing lines was significant at P<0.01. Interestingly, the decrease in stomatal number appeared to be partly due to an increase in cell size. Cells of transgenic lines appeared to be larger in size with fewer cells in the same relative area. The decrease in non-stomatal cell number was about 25% in transgenic *SlERF36* over-expressing lines and those over-expressing *SlERF36ΔEAR* (Fig. 6C).

## Discussion

SlERF36 is a repressor type of ERF containing a C-terminal EAR motif that was shown to affect flowering time and senescence in transgenic tobacco plants [22]. Another prominent phenotype in tobacco was a 25–35% reduction in stomatal density that in turn reduced stomatal conductance, CO_2_ uptake and utilization and photosynthesis thereby affecting development. These could either be independent effects or they could be related. In order to elucidate the role of the EAR motif in these different changes, we generated transgenic *Arabidopsis* over-expressing the complete *SlERF36* and a truncated version of the gene lacking the EAR motif. Interestingly, all the effects observed in transgenic tobacco namely early flowering, early senescence, reduced stomatal number and reduced photosynthesis were replicated in transgenic *Arabidopsis* upon over-expression of the full-length *SlERF36*. The observations have two major implications:

1. The targets of SlERF36 action for early flowering, early senescence and stomatal number are most likely conserved between tomato/tobacco and *Arabidopsis* suggesting functional conservation of these pathways.
2. The sites for SlERF36 binding, upstream within the promoter of the target genes, are also most likely conserved between *Arabidopsis* and tomato/tobacco for it to bind and produce similar effects in these plants.

The EAR motif has been associated with active repression of transcription by several repressor genes that function in different processes [27] and its presence is necessary for repression since mutations in the EAR motif reduce or abolish the repression activity and gene function [4], [10]. For instance, plants expressing the Arabidopsis *RAP2.1* show enhanced sensitivity to drought while mutation of the EAR motif reduces drought sensitivity [16]. Similarly, plants expressing *SlERF3* show severe growth suppression while plants expressing the gene without the EAR motif have no suppressive effect on growth [17]. Likewise, in tobacco, induction of hypersensitive cell death expression by *NtERF3* is dependent on the EAR motif [28]. In another study, transgenic rice expressing a mutated version of the EAR motif of the *OsERF3* gene, showed increased ethylene biosynthesis and greater drought tolerance compared to the non-mutated *OsERF3* expressing lines that showed suppression of ethylene biosynthesis genes [19]. In some cases however, complete removal of the EAR motif does not affect all aspects of the gene function. For example, expression of *ZAT7* reduces growth and imparts salt tolerance but deletion of the EAR motif from ZAT7 does not reduce growth suppression although plants lose their salinity tolerance [29].

Of the changes that we observed, the most prominent visible change was the early flowering phenotype. Interestingly, early flowering was seen not only under long day conditions (both 16 h light and 24 h light) but also under short day conditions (10 h light) indicating that the effects on flowering were largely photoperiod independent. Nevertheless, it should be noted that transgenic plants grown under short day conditions did show a delay in flowering compared to those grown under long day conditions indicating that photoperiod did to some extent influence the timing of flowering in transgenic lines. The early flowering was associated with higher levels of the *FT* (*FLOWERING LOCUS T*) transcript, the gene involved in initiating flowering [30]. Expression of the truncated form of *SlERF36* (lacking the EAR motif) abrogated the early flowering phenotype of full length *SlERF36* expression under both short day and long day conditions. Its expression did not affect (increase) *FT* transcript levels (Fig. 2D) in spite of the presence of the AP2 domain. This indicated that the presence of the EAR motif was essential for the higher *FT* transcript levels and the early flowering phenotype although an effect of other deleted C-terminal residues cannot be ruled out. The fact that *SlERF6* over-expression accelerates flowering regardless of photoperiod and in plants as different as *Arabidopsis* and tobacco suggests that SlERF36 (and the EAR motif) might interact in some way with the general flowering machinery and regulate a component that is common to both photoperiods. Considering that EAR motif containing proteins function as active repressors of transcription, and that *SlERF36* expression leads to increased *FT* transcript levels, one could envisage a possibility where the direct or indirect repression of a floral inhibitor by SlERF36 could activate *FT* and thereby flowering in the transgenic lines. An interesting possibility that would require further studies is whether SlERF36 affects expression of homologues of *TEMPRANILLO* (*TEM1* and *TEM2*) that are known to directly repress *FT* expression [31] or whether it in some way controls TOE1/TOE2 or miRNA172, the regulation of which affects flowering in both short and long day conditions [32]. Incidentally, both TEM and TOE members belong to the AP2/ERF/RAV domain family of transcription factors.

Of the other changes, those related to early senescence appeared to be a consequence of the early flowering phenotype and therefore developmental in nature. This is based on the observations that although senescence was early in transgenic *SlERF36* plants (as seen through reduced chlorophyll and higher expression of the *SEN4* gene), it was dependent on the photoperiodic flowering and was delayed when flowering was delayed in short day conditions. Under these conditions, rosette sizes were larger than under long day conditions and plants took a longer time to senesce (Fig. 2E). The reduced sizes of the various organs (rosettes, flowers and siliques) in transgenic *SlERF36* over-expressing plants under long day conditions was most likely an effect due to reduced vegetative growth of these plants and the fewer number of leaves that were present at the time of flowering, leading to fewer photosynthates being synthesized and translocated. In transgenic *SlERF36ΔEAR* plants, where the timing of flowering was not affected, senescence was also normal.

In contrast to the flowering and senescence phenotypes, the other major phenotype namely stomatal number was affected in both transgenic *SlERF36* plants and those lacking the EAR motif (albeit to a lesser extent). At least in plants over-expressing *SlERF36ΔEAR* the apparent reduction in stomatal number by 25% could be attributed to an increase in cell size which increased by about 25%. Stomatal density is known to be tightly controlled by a large number of negative regulators such as ERECTA, ERL1, ERL2, EPF1, EPF2, CHALLAH, YODA, TMM, and SDD1 that are responsible for determining the spacing between stomata [33]. The fact that SlERF36 reduces stomatal density by 25–35% in tobacco and 38–39% in *Arabidopsis* would indicate that a stomatal development regulator common to both tobacco and Arabidopsis might be controlled by the repressor SlERF36. Stomatal density is also controlled by environmental factors such as CO_2_, humidity, light intensity and water availability with CO_2_ levels being by far the most important determinants of stomatal density and conductance in angiospermic plants. An inverse relationship between CO_2_ levels and stomatal density and conductance has been noted under experimental conditions as well as in fossil studies [34], [35], [36] with CO_2_ doubling leading to an average decrease of almost 22–29% in stomatal density in *Arabidopsis* and other plants [37], [38]. Changes in stomatal density can affect photosynthesis and growth [39] particularly under conditions where there is no corresponding increase in CO_2_ levels. Indeed the reduction in stomatal number and density does affect photosynthesis with growth being affected in *SlERF36* over-expressing plants but not in *SlERF36ΔEAR* over-expressing plants (where the reduction in stomatal number is lower). Several studies in Arabidopsis have shown that reduced stomatal densities in the range of 20–25% such as in mutants like *gtl1, edt1, gpa1* do not affect plant growth [40], [41], [42] unlike in *SlERF36* expressing plants where a much a larger decrease is observed. It is likely that beyond a certain point the reduced density may limit CO_2_ availability affecting plant growth. Whether the effects on stomatal density/photosynthesis and flowering are related or independent effects is not yet known. Photosynthetic rates control starch reserves and these in turn could affect C/N ratios and thereby flowering. SlERF36 has recently been shown to actively repress the ethylene responsive GCC box *in vitro* [13]. The net effect of such a function would be to reduce ethylene responses. In this context, it is interesting to note that recent microarray studies in *SlERF36* expressing plants, although not conclusive, showed reduction in transcript levels of *AtERF1, AtERF2* (involved in ethylene responses) and *AtMKK9* (involved in ethylene biosynthesis), suggesting a reduction in ethylene responses (data not shown). The net effect of a reduction in ethylene responses would be an increase in cell size since ethylene is known to repress cell elongation. Ethylene is also known to delay flowering. Both these effects of increased cell size and early flowering, possibly indicative of reduced ethylene responses, are seen in transgenic *SlERF36* over-expressing lines. However, more detailed studies especially through loss of SlERF36 function lines are required to get at a causal relationship between SlERF36, ethylene responses and the early flowering.

In conclusion, we demonstrate that the EAR motif of SIERF36 is most likely responsible for the strong early flowering phenotype and a reduction in stomatal density and photosynthesis that is common to both Arabidopsis and tobacco when SlERF36 is over-expressed. The indication that this motif may also directly or indirectly control the expression of *FT*, although not studied in detail as yet by us, adds a new dimension to the complex pathways by which flowering is controlled in plants.
